# Isolation of archaeal viruses with lipid membrane from Tengchong acidic hot springs

**DOI:** 10.3389/fmicb.2023.1134935

**Published:** 2023-03-29

**Authors:** Xi Feng, Yanan Li, Chang Tian, Wei Yang, Xinyu Liu, Changyi Zhang, Zhirui Zeng

**Affiliations:** ^1^Department of Ocean Science and Engineering, Southern University of Science and Technology, Shenzhen, China; ^2^Carl R. Woese Institute for Genomic Biology, University of Illinois at Urbana-Champaign, Urbana, IL, United States; ^3^Southern Marine Science and Engineering Guangdong Laboratory, Guangzhou, China

**Keywords:** archaeal virus, *Sulfolobaceae*, hot springs, Tengchong, viral membrane lipid

## Abstract

Archaeal viruses are one of the most mysterious parts of the virosphere because of their diverse morphologies and unique genome contents. The crenarchaeal viruses are commonly found in high temperature and acidic hot springs, and the number of identified crenarchaeal viruses is being rapidly increased in recent two decades. Over fifty viruses infecting the members of the order *Sulfolobales* have been identified, most of which are from hot springs distributed in the United States, Russia, Iceland, Japan, and Italy. To further expand the reservoir of viruses infecting strains of *Sulfolobaceae*, we investigated virus diversity through cultivation-dependent approaches in hot springs in Tengchong, Yunnan, China. Eight different virus-like particles were detected in enrichment cultures, among which five new archaeal viruses were isolated and characterized. We showed that these viruses can infect acidophilic hyperthermophiles belonging to three different genera of the family *Sulfolobaceae*, namely, *Saccharolobus*, *Sulfolobus*, and *Metallosphaera*. We also compared the lipid compositions of the viral and cellular membranes and found that the lipid composition of some viral envelopes was very different from that of the host membrane. Collectively, our results showed that the Tengchong hot springs harbor highly diverse viruses, providing excellent models for archaeal virus-host studies.

## Introduction

Like bacteriophages (bacterial viruses), archaeal viruses are also one of the most abundant and diverse biological entities on Earth. Viruses of archaea shared very few features with those of bacteria or eukaryotes in that they exhibited exceptionally diverse morphologies, remarkably distinct genomic contents, and unique infection mechanisms. For example, the Sulfolobus spindle-shaped viruses (SSVs) represent one of the most well-studied virus prototypes infecting crenarchaeal hosts and the spindle-shaped viral particle morphology has only been observed in Archaea (Fulton et al., 2009). In terms of virion morphology, studies on archaeal viruses showed the morphological diversity of hyperthermophilic archaeal viruses appears to exceed that of viruses of prokaryotes (Baquero et al., 2020a; Wirth et al., 2021). Because the genomes of hyperthermophilic archaeal viruses could have predated cellular life according to the analysis results of comparative genomics, they serve as model systems for understanding the evolution of life (Prangishvili and Garrett, 2005).

Over one hundred of archaeal viruses have been discovered to date and these archaeal viruses are currently classified into 33 families (Liu et al., 2021). Mainly because of their relative ease of culturing and ubiquitous presence in high temperature and acidic environments, over 60 viruses from hyperthermophilic archaea of the genera *Acidianus*, *Metallosphaera,* and *Sulfolobus* have been identified by culture-independent approaches (Baquero et al., 2020b; Fackler et al., 2022; Han et al., 2022). To date, the viruses of the genus *Sulfolobus* are the best studied of the archaeal viruses and about a dozen of viruses have been identified from this genus (Prangishvili, 2003). Most viruses infecting *Sulfolobus* are found to be enveloped with few exceptions such as the well-studied Sulfolobus islandicus rod-shaped viruses (SIRVs) belonging to the family *Rudiviridae* (Lawrence et al., 2009). Like other archaeal viruses, the lipids of *Sulfolobus* viruses are composed of an isoprenoid ether lipid rather than the fatty acid ester lipids found in bacterial membranes (Sprott et al., 1991; Koga and Morii, 2007). However, the lipid composition of some *Sulfolobus* viral envelopes has been found to be quantitatively very different from that of the host membranes, even if the membrane lipids of all archaeal viruses are derived from the host lipid pool. For instance, Sulfolobus filamentous virus 1 (SFV1) and *Sulfolobus islandicus* filamentous virus (SIFV) were shown to selectively acquire host membrane lipids GDGT-0 (glycerol dibiphytanyl glycerol tetraether) or archaeol (Liu et al., 2018; Baquero et al., 2021a), yet the archaeol and GDGT-0 account for a small proportion of lipids in the host membrane and the dominant lipid species are tetraether lipids carrying four cyclopentane rings (GDGT-4). The mechanism of membrane lipid selection in viruses, however, has yet to be largely explored.

Tengchong hot springs are located on the northeastern edge of Tibet-Yunnan geothermal zone, an active geothermal region in Yunnan Province, China. These hot springs are known for the hot and acidic environmental conditions, and microbial communities in these hot springs have been found to be dominated by *Euryarchaeota* and *Crenarchaeota* (Pagaling et al., 2012; Jiang et al., 2016). Compared with the extensive studies of archaeal viruses isolated from hot springs in Yellowstone National Park (YNP) of United States (Rice et al., 2001), Umi Jigoku of Japan (Liu et al., 2019), and Campi Flegrei volcano of Italy (Baquero et al., 2020b), archaeal viruses from Tengchong hot springs, China, have been poorly explored. Particularly, the diversity of viruses infecting *Sulfolobus* has not been systematically investigated though it has been reported that the species of the family *Sulfolobaceae* are one of the most abundant archaea in Tengchong hot springs (Pagaling et al., 2012). To date, only two single-tailed fusiform viruses STSV1 and STSV2 from Tengchong hot spring have been isolated (Xiang et al., 2005, p. 1; Erdmann et al., 2013, p. 2). STSV1 and STSV2, sharing 80% identity at nucleotide sequence level, are double-stranded DNA viruses and enveloped by a lipid membrane structure. In this study, we aimed to explore virus diversity in Tengchong hot springs by using a cultivation-dependent approach. We isolated and characterized general features (such as virion morphology, genome contents, etc.) of five new archaeal viruses infecting *Sulfolobus* species, with focusing on the analysis of membrane lipid composition of viral envelope.

## Materials and methods

### Enrichment cultures

Eleven environmental samples were collected from the hot springs of Tengchong in Yunnan, China (Supplementary Table S1), and 1 g of each sediment sample was inoculated into 100 ml of rich medium favoring the growth of *Sulfolobus/Saccharolobus/Metallosphaera*. The rich medium was derived from the Brock medium (Allen, 1959; Brock et al., 2004) with proper modifications. It consisted of basal salt solution [1.3 g l^−1^ (NH_4_)_2_SO_4_, 0.28 g l^−1^KH_2_PO_4_, 0.25 g l^−1^ MgSO_4_·7H_2_O, 0.07 g l^−1^ CaCl_2_·2H_2_O, 1.8 mg l^−1^ MnCl_2_·7H_2_O, 4.5 mg l^−1^ Na_2_B_4_O_7_·107H_2_O, 0.22 mg l^−1^ ZnSO_4_·7H_2_O, 0.05 mg l^−1^ CuCl_2_·2H_2_O, 0.0 3 mg l^−1^ Na_2_MoO_4_·2H_2_O, 0.03 mg l^−1^ VOSO_4_·n7H_2_O, 0.01 mg l^−1^ CoSO_4_·7H_2_O], 0.2% (wt/vol) sucrose, 0.1% (wt/vol) tryptone, 0.1% (wt/vol) yeast extract, and 0.1% (wt/vol) amine. The pH was adjusted to 3.0 with 10 N H_2_SO_4_. The environmental samples were incubated for 10 days at 75°C with 200 rpm shaking.

### Virus-like particles concentration

To observe the virus-like particles (VLPs), the enrichment cultures were centrifuged at 6, 500 × *g* for 15 min at 15°C. The supernatant was filtered through a 0.22-μm filter (Merck Millipore), and the VLPs were precipitated from the supernatant by the addition of NaCl to 1 M and PEG 6000 to 10% (wt/vol) and incubated overnight at 4°C. The pellet was collected by centrifugation at 12,000 × *g* for 30 min at 4°C, and then suspended in 100 μl SM buffer containing: 0.1 M NaCl, 8 mM MgSO_4_·7H_2_O, 50 mM Tris–HCl, 0.005% (wt/vol) glycerol, pH 6.0 (Colombet et al., 2008).

### Electron microscopy

Negatively stained cells were prepared for transmission electron microscopy (TEM) by spotting 10 μl of culture or viral solutions onto carbon copper grids (KTKY, 400 mesh) for 2–5 min and air-dried. Samples were stained with 2% (wt/vol) uranyl acetate for 30 s, and then observed under an electron microscope (Thermo Fisher Scientific, Talos 120C) operated at 120 kV.

### Isolation and purification of viruses

The hot spring sediments were diluted with liquid Brock medium. Serial dilutions were directly spread onto plates’ medium supplemented with 1.0% Gelrite (wt/vol) and incubated under aerobic conditions for 14 days at 75°C. After incubation, single colonies were picked and inoculated onto plates by scribing to obtain purified strains. Then the purified strains were incubated and prepared for plaque assay. The cell culture was incubated for 1 h at 75°C to allow the adsorption of the virus to the host cells. Immediately the 600 μl cultures mixed with 4.4 ml modified Brock’s medium and Gelrite with final concentration 1.0%, and then poured onto the prepared bottom layer with modified Brock’s medium and 1.0% Gelrite. The plates were incubated for 3–5 days at 75°C to allow plaques to form. Zones of inhibition were cut out from the Gelrite plates and inoculated into exponentially growing cultures of the corresponding isolates. After incubation, the culture supernatant was examined by TEM. Meanwhile, pure viral strains were obtained by three rounds of single-plaque purification.

### 16S rRNA phylogeny

16S rRNA gene was amplified using archaeal-specific primers 0023aF (5′-CTCCGGTTGATCCTGCC-3′) and 1406R (5’-GACGGGCGGTGTGTGC-3′) (Rice et al., 2001). Sanger sequencing of amplicons with 1,383-bp in length was performed by the Tsingke Biotechnology Co., Ltd. (Guangzhou, China). The 16S rRNA gene sequence of strains was preliminarily identified by searching for matches in the EzBioCloud databases and NCBI databases.

### Viral DNA extraction, sequencing, and genome analysis

Purified viral particles were collected, and viral DNA was extracted with the MiniBEST Viral RNA/DNA Extraction Kit (TaKaRa, Ver.5.0). Viral DNA was sequenced by using the Illumina HiSeq-PE150 system. An Illumina shotgun library using the Illumina TruSeq Nano DNA Sample Prep Kit was reconstructed and sequenced in paired ends 150 bp × 2 using the Illumina HiSeq platform. Raw sequencing data were generated by Illumina base-calling software CASAVA v1.8.2^1^ according to its corresponding instructions. The sequenced reads were assembled using SPAdes 3.13.0 software (Bankevich et al., 2012). The remaining gaps of draft genomes were closed by sequencing PCR products.

Open reading frames (ORFs) were predicted on the assembled genomes using a combination of Prodigal (Hyatt et al., 2010), Geneious (Cooper et al., 2012), OrfFinder (Sayers et al., 2021) and manual curation. Their start positions were determined by selecting the largest possible ORFs with one of the three start codons (ATG, GTG, and TTG). The genome and translated ORFs were used to search the NCBI RefSeq database for similarity using BLASTn, BLASTx, and BLASTp. HHpred (Söding et al., 2005) and Phyre2 (Kelley et al., 2015) were also used to search for similarities to translated ORFs.

Multilocus sequence analysis was performed for all viral genomes. The phylogenetic analysis of viral whole genomic and proteome sequences was carried out by the VICTOR online resource, using the distance formula D6 (Meier-Kolthoff and Göker, 2017).

### Lipid extraction and analyses

For lipid analyses, virions were first precipitated and collected by the addition of NaCl to 1 M and PEG 6000 to 10% (wt/vol), and then purified by CsCl density gradient (1.3, 1.5 and 1.7 g/cc) ultracentrifugation (Beckman MLA-55, 240, 000 × g, 2 h, 15°C). The opalescent virus-containing band was collected, pelleted, and resuspended in the SM buffer (Nasukawa et al., 2017). The virus-free hosts were obtained by through serial rapid passages and host cells were collected by centrifugation at 10,000 × *g* for 5 min at 4°C. The cellular and viral lipids were hydrolyzed with 5 ml hydrochloric acid (HCl): methanol (MeOH) =1:9 (vol/vol) in 40 ml capped glass vials at 70°C for 8 h. After acid hydrolysis, 10 ml of dichloromethane (DCM) and 10 ml of Nanopure water were added to the mixture, and lipids were fully extracted twice by vortexing with another 5 ml of DCM in a 50-mL Teflon tube. After centrifugation for 5 min at 2, 800 × g to separate the aqueous and organic phases, the bottom organic phase layer was transferred to a new collection glass vial. Total extracts of 20 ml organic phases were combined and filtered through a 0.45-μm PTFE filter and dried by N_2_ gas flow.

Lipid analysis was performed on an Agilent 1,290 Infinity II high-performance liquid chromatography (HPLC) system coupled with Agilent 6,495 quadrupole mass spectrometer under positive ionization mode with an atmospheric pressure chemical ionization (APCI) source. Compounds were separated *via* two BEH HILIC columns (2.1 × 150 mm, 1.7 μm; Waters) in tandem at 30°C. The flow rate was 0.2 ml/min and the total run time was 120 min and 10 μl of each sample was injected. The elution gradient was set with solvent A of n-hexane and solvent B of n-hexane: iso-propanol = 9:1 (vol: vol). Compounds were eluted with 18% B in the first 25 min, and then linearly increased to 35% B in the next 25 min, and subsequently 100% B in another 30 min. Finally, the gradient returned to 18% B in 10 min and re-equilibrated for 30 min. APCI settings with parameters as shown below: Nebulizer pressure 60 psi, vaporizer temperature 400°C, drying gas (nitrogen) flow 6 l/min at 200°C, capillary voltage 3 kV, and corona 5 μA. Archaeal ether lipids were detected using a single ion monitoring (SIM) mode (m/z: 653, 1,304, 1,302, 1,300, 1,298, 1,296, 1,294, 1,292, 1,290, 1,288, and 1,286).

## Results

### Diversity of virus-like particles in enrichment cultures

**Figure 1:**
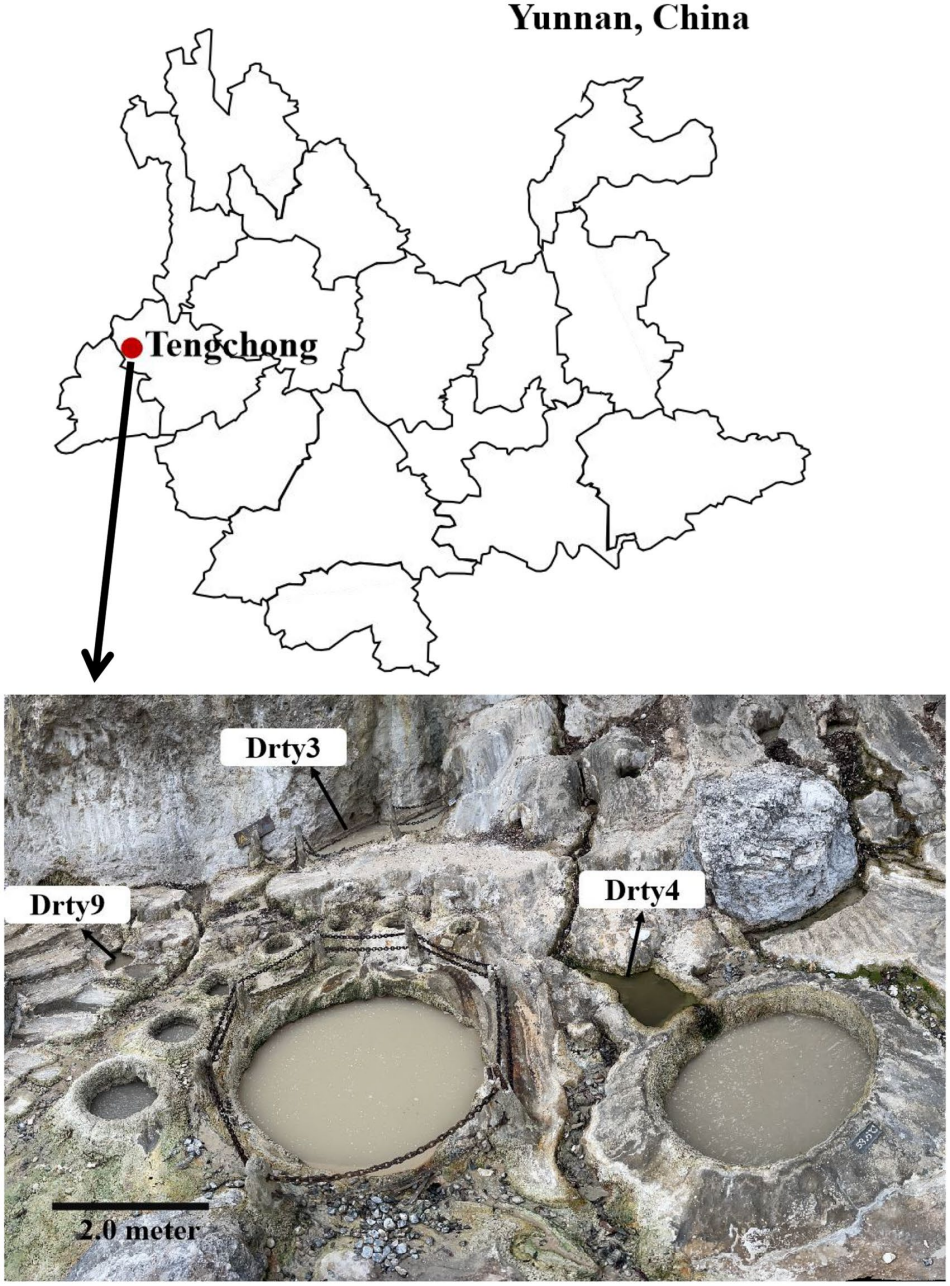
Location of Tengchong (red solid circle) in Yunnan Province, southwestern China, and the distribution of hot springs. Scale bar, 2 m.

**Figure 2:**
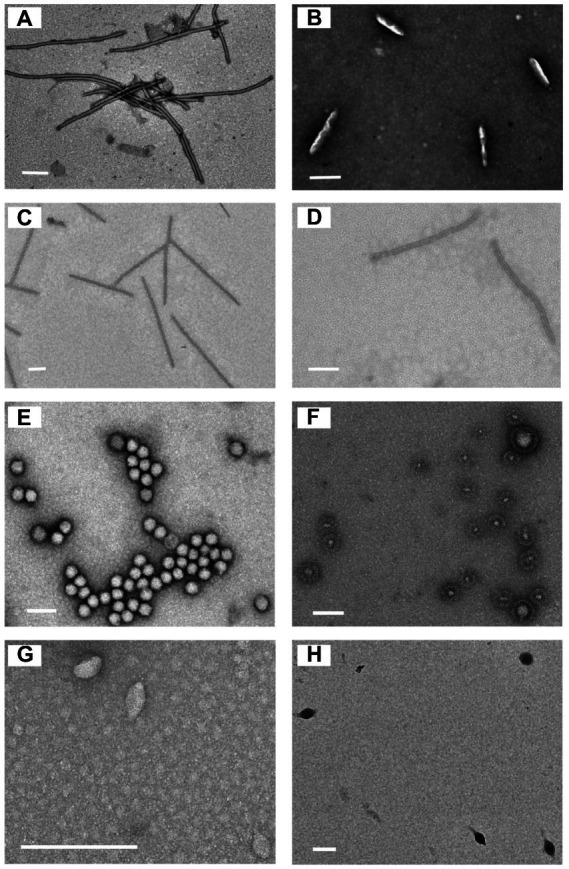
Transmission electron micrographs of the VLPs observed in enrichment cultures. **(A)** filamentous VLPs (870 ± 10 × 25 ± 2 nm); **(B)** short rod-shaped VLPs (220 ± 10 × 45 ± 5 nm, probably structural variants of spindle-shaped viruses); **(C)** thin rod-shaped VLPs (920 ± 20 × 20 ± 5 nm). **(D)** rod-shaped VLPs (750 ± 50 × 25 ± 2 nm); **(E)** spherical VLPs (50 ± 10 nm); **(F)** spherical enveloped VLPs (100 ± 90 nm, probably membrane vesicles); **(G)** spindle-shaped VLPs (55 ± 10 × 30 ± 12 nm); **(H)** tailed spindle-shaped VLPs (143 ± 20 × 85 ± 10 nm, tail 98 ± 10 nm). Scale bar, 200 nm.

Eleven sediment samples were collected from acidic hot springs and mud holes in Tengchong, Yunnan, China (Figure 1), with temperatures ranging from 56.3 to 92.0°C and pH values between 2.1 and 3.4 (Supplementary Table S1). The microbial diversity in the 11 samples was assessed by high-throughput sequencing of the PCR-amplified 16S rRNA genes from genomic DNA extracted from each sample. The sequencing results showed that the members of family *Sulfolobaceae* were the one of most abundant, within which isolates of *Sulfolobus*, *Saccharolobus*, and *Metallosphaera* account for about 73.7–94.0% of microbial community (Supplementary Figure S1). Archaea, particularly hyperthermophilic crenarchaea, have been shown to be the hosts of many viruses with diverse virion shapes and distinct genome compositions (Iranzo et al., 2016). These dominated organisms thus could be the major hosts for archaeal viruses in Tengchong acidic hot springs. Therefore, the modified Brock’s medium favoring the growth of *Sulfolobales* was used to establish enrichment cultures for each sample. After incubation for 10 days at 75°C, virus-like particles (VLPs) were collected from supernatants and analyzed by transmission electron microscopy (TEM). Eight different VLPs were detected in the nine enrichment cultures (Figure 2). Based on virion morphologies, the VLPs could be assigned to four types: filamentous virions, rod-shaped virions, spherical virions, and spindle-shaped virions. Although these nine hot spring samples are located close and share many common characteristics, we found that the morphologies and number of VLPs observed were significantly different even after cultivation with the same growth medium and conditions (Supplementary Table S2). Among them, three major types of virions, i.e., the filamentous (Figure 2A), polyhedral (Figure 2E), and tailed spindle-shaped (Figure 2H) virion were observed in the enrichment cultures of three samples Drty3, Drty4, and Drty9, which were further used to isolate VLP-propagating strains.

### Isolation and purification of virus-host pairs

To establish the virus-host relationship, strains obtained from the three samples Drty3, Drty4 and Drty9 were examined. Finally, 202 single strain isolates were obtained from the three samples. Five virus-carrying strains (1201–2, 1,201–4, D4-4, D4-HJ, and 1,201–1) belong to family *Sulfolobaceae* (Table 1), isolated by plating on solid plates, were found to carry morphologically different viruses. Comparison of their 16S rRNA gene sequences in the GenBank databases^2^ and phylogenetic tree building based on the 16S rRNA gene sequences revealed that the virus-carrying strains were phylogenetically close to three different genera of the acidophilic hyperthermophiles family *Sulfolobaceae*, namely, *Saccharolobus*, *Sulfolobus,* and *Metallosphaera* (Supplementary Figure S2). The 16S rRNA gene sequences of strains 1,201–2 and 1,201–4 displayed 100% identity for the pairwise comparison.

**Table 1:** The virus-host pairs in the study.

Virus	Host	Morphology	Size (nm)
STSV3	*Sulfolobus* sp. 1,201–2	Spindle-shaped with a tail	143 ± 20 × 85 ± 10, (tail 98 ± 10)
STSV4	*Sulfolobus* sp. 1,201–4	Spindle-shaped with two tails	204 ± 20 × 96 ± 5 (tails,103 ± 50)
MTIV3	*Metallosphaera* sp. D4-4	Spherical	55 ± 5
SIFV3	*Saccharolobus* sp. 1,201–1	Filamentous	927 ± 60 × 25 ± 5
STIV3	*Sulfolobus* sp. D4-HJ	Spherical	70 ± 10

**Figure 3:**
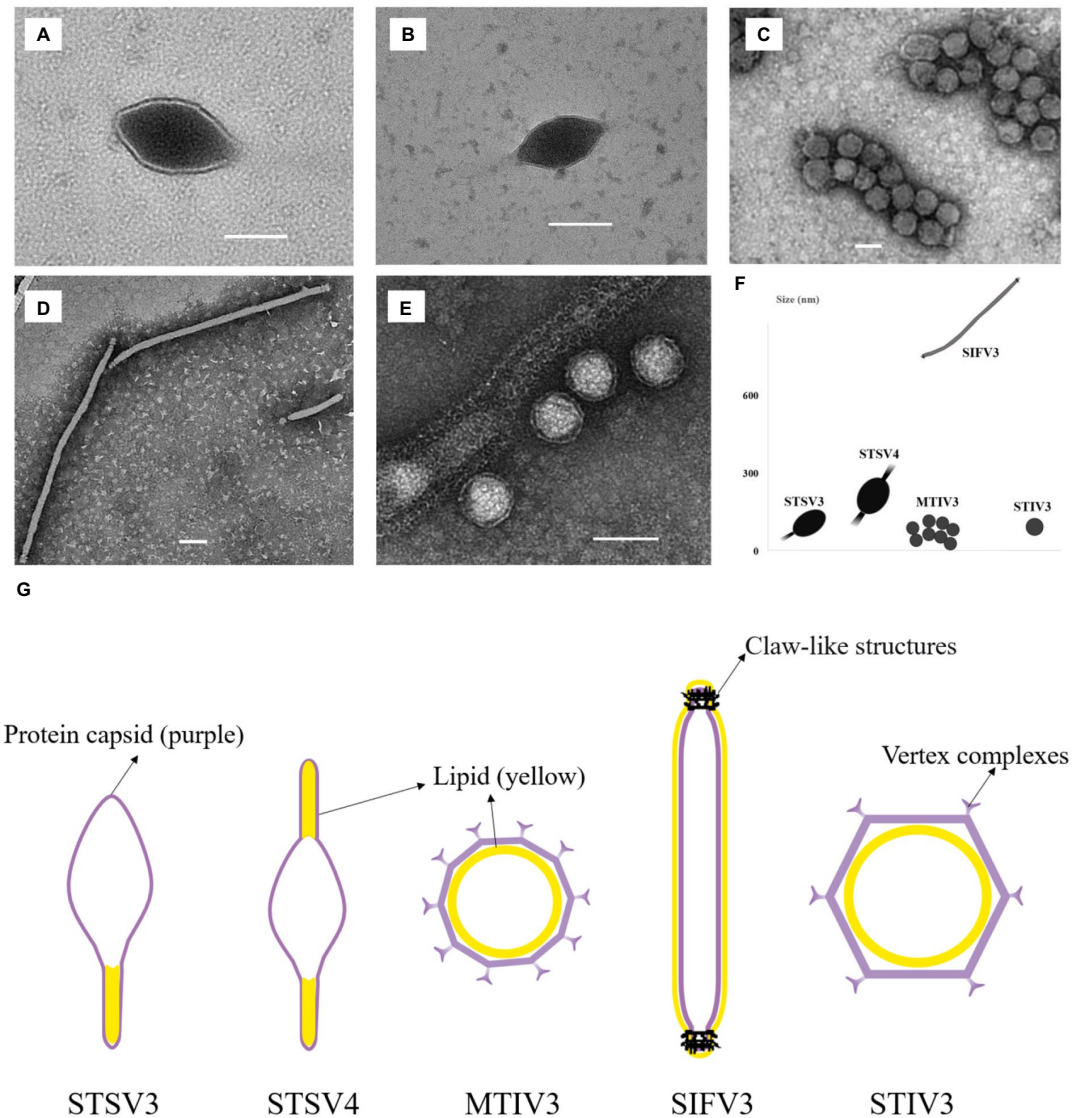
Transmission electron micrographs of the five isolated viruses in the study. **(A,B)** STSV3 and STSV4 (tailed spindle-shaped virions); **(C)** MTIV3 (spherical virions); **(D)** SIFV3 (filamentous virions); **(E)** STIV3 (spherical enveloped virions); **(F)** The morphology and size of these viruses. **(G)** Schematic representation of the five viruses with the lipid membrane (Reuter et al., 2005; Wagner et al., 2017; Han et al., 2022; Wang et al., 2022). The inside or outside membrane is illustrated as a yellow layer, and the protein capsid is depicted in purple. **(A–E)** Scale bar, 100 nm. The viral particles are not drawn in scale in **(G)**.

Two spindle-shaped viruses that can infect *Sulfolobus* sp. 1,201–2 and *Sulfolobus* sp. 1,201–4 are found, both of which displayed 99% identity to the corresponding 16S rRNA gene sequences of *Sulfolobus tengchongensis* RT8-4. One virus, temporarily named as STSV3 (*Sulfolobus tengchongensis* spindle-shaped virus 3), has a spindle-shaped morphology (143 ± 20 × 85 ± 10 nm) with a tail of variable length (98 ± 10 nm) at one end (Figure 3A). Another tailed spindle-shaped virus, temporarily named as STSV4, has a relatively larger size (204 ± 20 × 96 ± 5 nm with 103 ± 50 nm tails) compared to that of STSV3. Different from STSV1 and STSV2 which have a single short tail positioned at only one of the two similar poles, viral particles of STSV4 are morphologically similar to ATV (Acidianus two-tailed virus) (Prangishvili et al., 2006) and have tails at each pointed end (Figure 3B). The tail fibers of these fusiform viruses are probably involved in host recognition (Lawrence et al., 2009). We also discovered a virus with spherical viral particles, which was temporarily named as Metallosphaera turreted icosahedral virus 3 (MTIV3) and found to be able to propagate in *Metallosphaera* sp. D4-4 which displayed 99% identity to the 16S rRNA gene sequence of *Metallosphaera prunae* Ron12/II (Fuchs et al., 1996; Figure 3C). Negatively stained virions of MTIV3 showed they are spherical particles of around 50 ± 5 nm of diameter with vertex complexes (Figure 3G). The filamentous virion infecting *Saccharolobus* sp. 1,201–1, which displayed 99% identity to the 16S rRNA gene sequence of *Saccharolobus shibatae* B12, was temporarily named as Saccharolobus shibatae filamentous virus 3 (SIFV3) (Figure 3D). Like other viruses belonging to family *Lipothrixviridae*, the ~927 nm long filamentous virions exhibited claw-like structures at both ends of the virions (Figure 3D) that are similar to what has been reported for AFV2 (Reuter et al., 2005). Another virus with spherical-shaped virions, temporarily named as Sulfolobus turreted icosahedral virus 3 (STIV3; Figure 3E), was found to infect *Sulfolobus* sp. D4-HJ (Table 1). Negatively stained virions of STIV3 showed that they are spherical particles of around 70 ± 5 nm of diameter (Figure 3E). The five viruses varied in morphology and size (Figure 3F).

### Analyses of viral genomes

The five viral DNA was extracted and sequenced on the Illumina MiSeq platform, with the assembled contigs corresponding to three complete virus genomes (SIFV3, MTIV3, and STIV3) and two incomplete virus genomes (STSV3 and STSV4). The genomes of new filamentous virus (SIFV3) and spherical viruses (MTIV3 and STIV3) show low similarity (<1%) on the nucleotide sequence level to the sequences in the public databases. The genes of those viruses show little similarity to genes of known function. However, analysis of the protein sequences encoded in the viral genomes showed that some putative proteins were homologous. The genome of SIFV3 is 30,153 bp in length and contains 56 ORFs, of which 18 ORFs have homologs in rod-shaped or filamentous viruses of the order *Sulfolobales* including three conserved lipothrixviral proteins, two virion structural proteins, and a glycosyltransferase (Supplementary Table S3), suggesting that SIFV3 is a *bona fide* member of the family *Lipothrixviridae*. The MTIV3 genome is 12,735 bp in length and encodes 33 putative ORFs. Homology searches using the BLASTp program revealed that only seven ORFs are significantly similar (E-value cutoff of 1e-03) to sequences in the non-redundant protein database and six of these ORFs have homologs in MTIVs (Metallosphaera turreted icosahedral virus) (Wagner et al., 2017; Supplementary Table S3). The STIV3 genome is 18,040 bp in length and encodes 47 putative ORFs, of which 13 ORFs show significant similarities to sequences in the database. Eight of these predicted proteins share homologs exclusively with MTIVs and ORF33 is highly similar (61.9% identity at the amino acid level) to the structural protein c137 of MTIVs (Supplementary Table S3). Although the genomes of both MTIV3 and STIV3 encode homologous proteins with MTIVs, the genome sequence of STIV3 and MTIV3 shows no similarity, and only ORF21 of MTIV3 is similar (48.3% identity at the amino acid level) to the ORF33 of STIV3. The incomplete STSV3 genome is 77,497 bp in length and contains 90 predicted ORFs. The incomplete STSV4 genome is 54,683 bp in length and contains 66 predicted ORFs (Table 2). The incomplete genome of STSV3 showed high similarity on the nucleotide sequence level (85 ~ 90.2% identity) with STSV2 (genome size 76,107 bp), and STSV4 also showed 80 ~ 86% identity on the nucleotide sequence level with STSV1 (genome size 75,294 bp).

**Table 2:** Genomic properties of the novel viruses from this study.

Virus	Genome size (bp)	Contigs	GC%	ORF	Accession number
STSV3	72,061	7	35	88	OP999020-OP999026
STSV4	43,181	11	36.2	66	OP999027-OP999037
MTIV3	12,735	1	51.1	33	OQ473814
SIFV3	30, 153	1	39.6	56	OQ473815
STIV3	18,040	1	42.4	47	OQ473816

**Figure 4:**
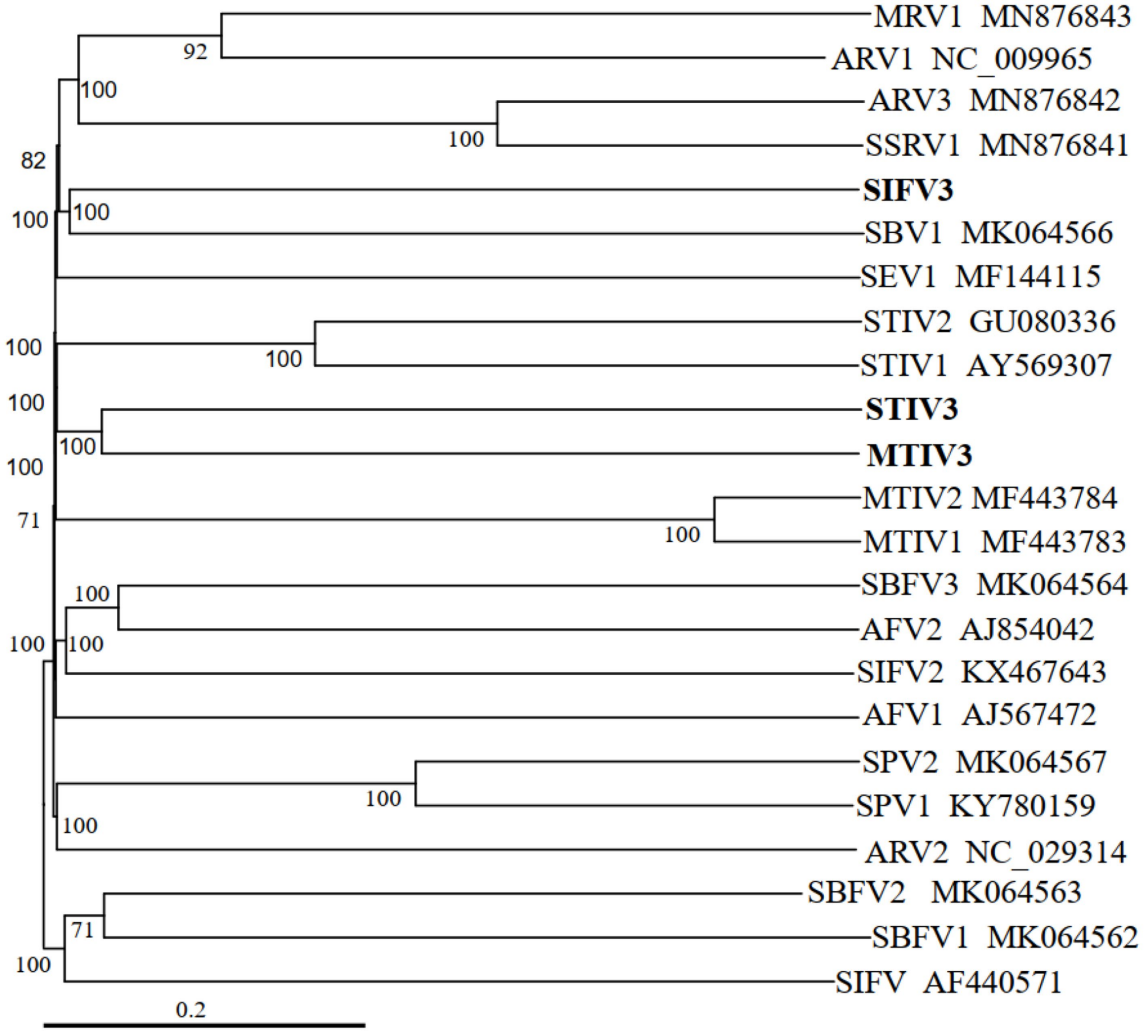
Phylogenetic tree of archaeal viruses based on whole genome analysis at the amino acid level using VICTOR (Meier-Kolthoff and Göker, 2017). The tree is rooted at mid-point, and the branch length is scaled in terms of the distance formula D6. The branch supporting value >50% is shown. For each genome, the virus name and the GenBank accession number are indicated. The newly isolated viruses from this study are marked in bold font.

Phylogenetic tree of archaeal viruses shows the evolutionary positions of the newly isolated viruses MTIV3, SIFV3, and STIV3, as illustrated in Figure 4. The new filamentous virus SIFV3 is similar at the amino acid level to the reported filamentous viruses of *Sulfolobus* and together with the members of the family *Lipothrixviridae.* MTIV3 and STIV3 fall into the same clade and related with MTIVs (Metallosphaera turreted icosahedral virus) and STIVs (Sulfolobus turreted icosahedral virus) which are the known spherical dsDNA viruses (Figure 4). Notably, our results unequivocally show that the viruses isolated from Tengchong belong to different families.

### Archaeal virus membrane lipids

**Figure 5:**
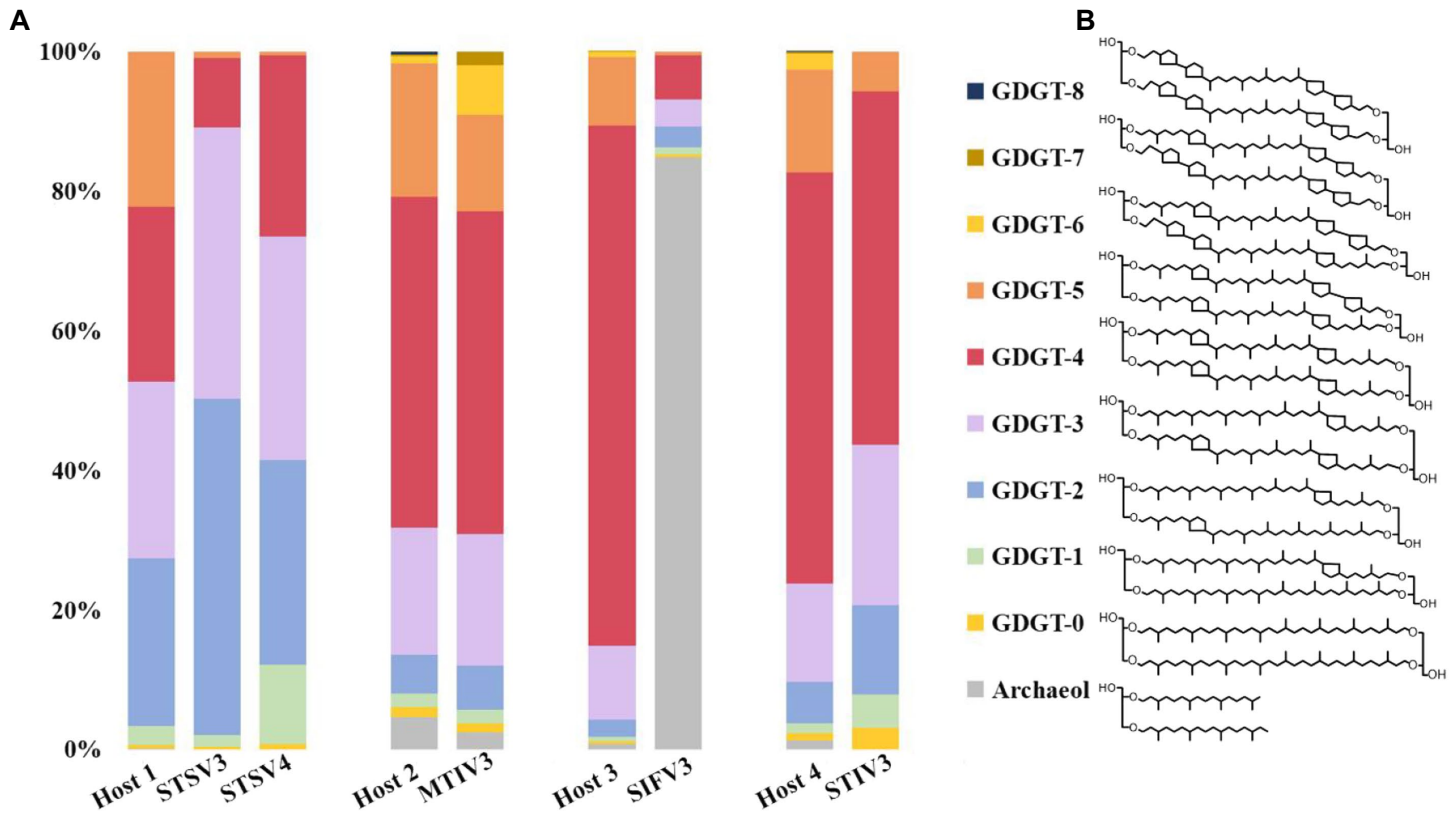
Distribution of lipid species identified in host cells and the highly purified virions. **(A)** The lipid compositions and the proportion of the virus envelope and host cellular membranes; **(B)** Chemical diagrams for the GDGT 0–8 (GDGT, glycerol dibiphytanyl glycerol tetraether) and archaeol. Host 1: *Sulfolobus* sp. 1,201–2/1201–4; Host 2: *Metallosphaera* sp. D4-4; Host 3: *Saccharolobus* sp. 1,201–1; Host 4: *Sulfolobus* sp. D4-HJ.

To determine and compare the lipid compositions of the virus envelope and host cellular membranes, liquid chromatography-mass spectrometry (LC–MS) was used to analyze lipids extracted from mature virions and host cells. The lipid composition of the filamentous SIFV3 was found to be substantially different from that of the host membrane (Figure 5). The *Saccharolobus* sp.1201–1 membrane is mainly composed of GDGTs, and particularly, the GDGTs with four cyclopentane rings (GDGT-4) are dominant. By contrast, the envelope of SIFV3 is strongly enriched in diether lipid archaeol. The lipid archaeol is less than 1% in the host membrane but reaches over 70% of lipids in the viral envelope. Thus, like other filamentous viruses AFV1, SFV1, and SIFV which selectively acquired archaeol or GDGT-0 from the pool of host lipids (Wang et al., 2019; Baquero et al., 2021a; Han et al., 2022), the lipids are incorporated into the SIFV3 envelope in a highly selective manner. The lipid compositions of the spindle-shaped virus STSV3 and STSV4 were slightly different from that of their host membrane. Particularly, GDGT-5 was one of the dominant lipids in the host membrane (∼20.2% of all ether lipids), but only small amount of GDGT-5 was found in STSV3 (∼0.7% of all ether lipids) (Figure 5). Recent structural studies suggested that lipids may be present in the termini of the spindle-shape virions (Figure 3G; Han et al., 2022; Wang et al., 2022). While, the lipid compositions of MTIV3 and STIV3 are similar to that of their hosts, suggesting that these viruses do not selectively acquire host membrane lipids (Figure 5).

## Discussion

We surveyed the microbial diversity in hot and acidic springs of Tengchong by high-throughput sequencing of the PCR-amplified 16S rRNA genes and found the members of family *Sulfolobaceae* were the one of most abundant. These dominated organisms thus could be the major hosts for the archaeal viruses in Tengchong hot springs. We also explored the virus diversity by establishing the enrichment culture and isolating crenarchaeal viruses that infect the members belonging to the family *Sulfolobaceae*. Finally, eight different particles were found in *Sulfolobus* enrichment cultures grown at 75°C and pH 3.0 for 10 days, and five viruses were isolated and identified. Two of the viral particle (STSV3 and STSV4) morphologies are similar to viruses infecting *Sulfolobus* species previously. Three viral particles (MTIV3, SFV3, and STIV3) morphologies have not been previously reported from Tengchong hot springs. The results showed there are many crenarchaeal viruses with diverse morphologies in the Tengchong hot spring, which probably play key roles to drive microbial evolution and shape population structure. The five isolated viruses further expand the reservoir of already existed crenarchaeal viruses and provide new resources to study archaeal virus-host interaction and co-evolution.

Despite some of virus-like particles isolated from Tengchong have nearly identical morphologies to MTIVs, SBFV3, and SPV1 isolated from Yellowstone National Park of United States and Beppu of Japan, the analysis of their genome sequences indicates that they are genetically distinct. The use of enrichment cultures was proved to be an effective way to isolate archaeal viruses in this study; however, this cultivation-dependent approach greatly limits the growth of other potential viral hosts as the culturing condition were selected in a bias way to support *Sulfolobus* growth. Thus, it is reasonable to assume that a large number of potential viral hosts belonging to other families exist in Tengchong hot springs. We envision that viral metagenome (virome) analysis will be powerful to explore the viral diversity in acidic hot springs of Tengchong; and the analysis of CRISPR (Clustered Regularly Interspaced Short Palindromic Repeats) spacer match between virus and host will gain some new insights into virus-host interactions.

Archaeal viruses represent one of the least known territories of the viral universe and even less is known about their lipids. Archaeal viruses can acquire host membrane lipids as their inner membrane or outside envelope. Notably, the virions of MTIV3 and STIV3 are likely surrounded by the inner lipid membranes and further encased by the outer polyhedral capsids (Figure 3G) as previously described on the icosahedral archaeal viruses SPV1, STIV, and STIV2, and MTIVs (Roine and Bamford, 2012; Wagner et al., 2017; Wang et al., 2019). It has been proposed that the viral inner lipid membrane lipids are synthesized *de novo* (Fu et al., 2010). Different from MTIV3 and STIV3 with inner membrane, SIFV3 has the outside envelope like SIFV (Baquero et al., 2021b; Figure 3G). STSV3 and STSV4 are tailed spindle-shaped viruses, which have been reported to have outside envelope (Owens et al., 2012), but latest Cryo-EM structure assays showed these viruses do not contain outside lipid membrane; instead, lipids are possible located in the termini of virus (Han et al., 2022; Wang et al., 2022; Figure 3G). In our study, we found that lipid composition of some viral inner membrane or outside envelopes was significantly different from that of the host membrane. For instance, the major membrane lipid composition of SIFV3 is substantially different from that of the host and the dominant lipid species in the viral membrane is archaeol. Similarly, the icosahedral virus SPV1 with inner membrane, the spindle-shaped viruses SSV1 and Sulfolobus monocaudavirus 1 (SMV1) with possible termini lipids, and the filamentous viruses (Acidianus filamentous virus 1 [AFV1], SFV1 and SIFV) with outside envelope could selectively acquire archaeol or GDGT-0 from the pool of host lipids (Quemin et al., 2015; Kasson et al., 2017; Liu et al., 2018; Wang et al., 2019; Baquero et al., 2021a; Han et al., 2022). To date, the mechanism of membrane lipid selection is still not be clearly explained. Our findings identified more archaeal viruses with different lipid profiles from their hosts, and we believed that the mechanistic studies of membrane lipid selection will be greatly facilitated in the future by establishing elegant genetic systems in archaeal viruses and their corresponding hosts.

## Data availability statement

The datasets presented in this study can be found in online repositories. The names of the repository/repositories and accession number(s) can be found in the article/Supplementary material.

## Author contributions

XF, CZ, and ZZ conceived and designed the experiments. XF performed most of the experiments. YL, WY, and XL helped with the virus isolation. XF wrote the draft with substantial input from CZ and ZZ. CZ and ZZ supervised the study. All authors contributed to the article and approved the submitted version.

## Funding

This work was supported by the National Natural Science Foundation of China (No. 32170041 and No. 92051112), the Science, Technology, and Innovation Commission of Shenzhen Municipality (No. 20200925154325002), and the Southern Marine Science and Engineering Guangdong Laboratory (Guangzhou) (No. K19313901).

## Conflict of interest

The authors declare that the research was conducted in the absence of any commercial or financial relationships that could be construed as a potential conflict of interest.

## Publisher’s note

All claims expressed in this article are solely those of the authors and do not necessarily represent those of their affiliated organizations, or those of the publisher, the editors and the reviewers. Any product that may be evaluated in this article, or claim that may be made by its manufacturer, is not guaranteed or endorsed by the publisher.

## Footnotes

^1^ http://support.illumina.com/

^2^ https://www.ncbi.nlm.nih.gov

## References

[ref1] Allen M. B. (1959). Studies with cyanidium caldarium, an anomalously pigmented chlorophyte. Arch. Mikrobiol. 32, 270–277. doi: 10.1007/BF00409348, PMID: 13628094

[ref2] Bankevich A. Nurk S. Antipov D. Gurevich A. A. Dvorkin M. Kulikov A. S.. (2012). SPAdes: a new genome assembly algorithm and its applications to single-cell sequencing. J. Comput. Biol. 19, 455–477. doi: 10.1089/cmb.2012.0021, PMID: 22506599 PMC3342519

[ref01] Baquero D. P. Liu Y. Wang F. Egelman E. H. Krupovic M. (2020a). Structure and assembly of archaeal viruses. Adv. Virus Res. 108, 127–164. doi: 10.1016/bs.aivir.2020.09.004 33837715

[ref3] Baquero D. Contursi P. Piochi M. Bartolucci S. Liu Y. Cvirkaite-Krupovic V.. (2020b). New virus isolates from Italian hydrothermal environments underscore the biogeographic pattern in archaeal virus communities. ISME J. 14, 1821–1833. doi: 10.1101/2020.01.16.907410, PMID: 32322010 PMC7305311

[ref4] Baquero D. P. Gazi A. D. Sachse M. Liu J. Schmitt C. Moya-Nilges M.. (2021a). A filamentous archaeal virus is enveloped inside the cell and released through pyramidal portals. Proc. Natl. Acad. Sci. U. S. A. 118:e2105540118. doi: 10.1073/pnas.2105540118, PMID: 34341107 PMC8364153

[ref5] Baquero D. P. Liu J. Prangishvili D. (2021b). Egress of archaeal viruses. Cell. Microbiol. 23:e13394. doi: 10.1111/cmi.13394, PMID: 34515400

[ref6] Brock T. D. Brock K. M. Belly R. T. Weiss R. L. (2004). Sulfolobus: a new genus of sulfur-oxidizing bacteria living at low pH and high temperature. Arch. Mikrobiol. 84, 54–68. doi: 10.1007/BF00408082 4559703

[ref7] Colombet J. Robin A. Lavie L. Bettarel Y. Cauchie H.-M. Sime-Ngando T. (2008). Virioplankton ‘Pegylation’: use of PEG (polyethylene glycol) to concentrate and purify viruses in pelagic ecosystems. J. Microbiol. Methods 71, 212–219. doi: 10.1016/j.mimet.2007.08.012, PMID: 17897741

[ref8] Cooper A. Kearse M. Meintjes P. Moir R. Wilson A. Stones-Havas S.. (2012). Geneious basic: an integrated and extendable desktop software platform for the organization and analysis of sequence data. Bioinformatics 28, 1647–1649. doi: 10.1093/bioinformatics/bts199, PMID: 22543367 PMC3371832

[ref9] Erdmann S. Chen B. Huang X. Deng L. Liu C. Shah S.. (2013). A novel single-tailed fusiform Sulfolobus virus STSV2 infecting model Sulfolobus species. Extremophiles 18, 51–60. doi: 10.1007/s00792-013-0591-z, PMID: 24163004

[ref10] Fackler J. Dworjan M. Gazi K. Grogan D. (2022). Diversity of SIRV-like viruses from a North American population. Viruses 14:1439. doi: 10.3390/v14071439, PMID: 35891419 PMC9319562

[ref11] Fu C. Y. Wang K. Gan L. Lanman J. Khayat R. Young M. J.. (2010). In vivo assembly of an archaeal virus studied with whole-cell electron cryotomography. Structure 18, 1579–1586. doi: 10.1016/j.str.2010.10.005, PMID: 21134637 PMC3042139

[ref12] Fuchs T. Huber H. Teiner K. Burggraf S. Stetter K. O. (1996). *Metallosphaera prunae*, sp nov, a novel metal-mobilizing, Thermoacidophilic archaeum, isolated from a uranium mine in Germany. Syst. Appl. Microbiol. 18, 560–566. doi: 10.1016/S0723-2020(11)80416-9

[ref13] Fulton J. Douglas T. Young A. (2009). Isolation of viruses from high temperature environments. Methods Mol. Biol. 501, 43–54. doi: 10.1007/978-1-60327-164-6_5 19066809

[ref14] Han Z. Yuan W. Xiao H. Wang L. Zhang J. Peng Y.. (2022). Structural insights into a spindle-shaped archaeal virus with a sevenfold symmetrical tail. Proc. Natl. Acad. Sci. 119:e2119439119. doi: 10.1073/pnas.2119439119, PMID: 35895681 PMC9351363

[ref15] Hyatt D. Chen G.-L. Locascio P. F. Land M. L. Larimer F. W. Hauser L. J. (2010). Prodigal: prokaryotic gene recognition and translation initiation site identification. BMC Bioinformatics 11:119. doi: 10.1186/1471-2105-11-119, PMID: 20211023 PMC2848648

[ref16] Iranzo J. Koonin E. Prangishvili D. Krupovic M. (2016). Bipartite network analysis of the archaeal virosphere: evolutionary connections between viruses and capsidless mobile elements. J. Virol. 90, 11043–11055. doi: 10.1128/JVI.01622-16, PMID: 27681128 PMC5126363

[ref17] Jiang Z. Li P. Jiang D. Dai X. Zhang R. Wang Y.. (2016). Microbial community structure and arsenic biogeochemistry in an acid vapor-formed spring in Tengchong geothermal area, China. PLoS One 11:e0146331. doi: 10.1371/journal.pone.0146331, PMID: 26761709 PMC4711897

[ref18] Kasson P. Dimaio F. Xiong Y. Lucas-Staat S. Egelman E. H. (2017). Model for a novel membrane envelope in a filamentous hyperthermophilic virus. Elife 22:e26268. doi: 10.7554/eLife.26268 PMC5517147 28639939

[ref19] Kelley L. Mezulis S. Yates C. Wass M. Sternberg M. (2015). The Phyre2 web portal for protein modeling, prediction and analysis. Nat. Protoc. 10, 845–858. doi: 10.1038/nprot.2015.053, PMID: 25950237 PMC5298202

[ref02] Koga Y. Morii H. (2007). Biosynthesis of ether-type polar lipids in archaea and evolutionary considerations. Micro biol. Mol. Biol. Rev. 71, 4. doi: 10.1128/MMBR.00033-06 PMC1847378 17347520

[ref20] Lawrence C. Menon S. Eilers B. Bothner B. Khayat R. Douglas T.. (2009). Structural and functional studies of Archaeal viruses. J. Biol. Chem. 284, 12599–12603. doi: 10.1074/jbc.R800078200, PMID: 19158076 PMC2675988

[ref21] Liu Y. Brandt D. Ishino S. Ishino Y. Koonin E. V. Kalinowski J.. (2019). New archaeal viruses discovered by metagenomic analysis of viral communities in enrichment cultures. Environ. Microbiol. 21, 2002–2014. doi: 10.1111/1462-2920.14479, PMID: 30451355 PMC11128462

[ref22] Liu Y. Demina T. A. Roux S. Aiewsakun P. Kazlauskas D. Simmonds P.. (2021). Diversity, taxonomy, and evolution of archaeal viruses of the class Caudoviricetes. PLoS Biol. 19:e3001442. doi: 10.1371/journal.pbio.3001442, PMID: 34752450 PMC8651126

[ref23] Liu Y. Osinski T. Wang F. Krupovic M. Schouten S. Kasson P.. (2018). Structural conservation in a membrane-enveloped filamentous virus infecting a hyperthermophilic acidophile. Nat. Commun. 9:3360. doi: 10.1038/s41467-018-05684-6, PMID: 30135568 PMC6105669

[ref24] Meier-Kolthoff J. P. Göker M. (2017). VICTOR: genome-based phylogeny and classification of prokaryotic viruses. Bioinformatics 33, 3396–3404. doi: 10.1093/bioinformatics/btx440, PMID: 29036289 PMC5860169

[ref25] Nasukawa T. Uchiyama J. Taharaguchi S. Ota S. Ujihara T. Matsuzaki S.. (2017). Virus purification by CsCl density gradient using general centrifugation. Arch. Virol. 162, 3523–3528. doi: 10.1007/s00705-017-3513-z, PMID: 28785814

[ref26] Owens F. Di Serio F. Li S. Pallás V. Randles J. (2012). Virus Taxonomy: Ninth Report of the International Committee on Taxonomy of Viruses. Amsterdam, Netherlands: Elsevier, 1221–1234.

[ref06] Pagaling E. Grant W. Cowan D. Jones B. Ma Y. Ventosa A.. (2012). Bacterial and archaeal diversity in two hot spring microbial mats from the geothermal region of Tengchong, China. Extremophiles: life under extreme conditions 16, 607–18. doi: 10.1007/s00792-012-0460-1 22622647

[ref27] Prangishvili D. (2003). Evolutionary insights from studies on viruses of hyperthermophilic archaea. Res. Microbiol. 154, 289–294. doi: 10.1016/S0923-2508(03)00073-1, PMID: 12798234

[ref28] Prangishvili D. Garrett R. A. (2005). Viruses of Hyperthermophilic Crenarchaea. Amsterdam, Netherlands: Elsevier, 535–542. 10.1016/j.tim.2005.08.013 16154357

[ref29] Prangishvili D. Vestergaard G. Reuter M. Aramayo R. Basta T. Rachel R.. (2006). Structural and genomic properties of the hyperthermophilic archaeal virus ATV with an extracellular stage of the reproductive cycle. J. Mol. Biol. 359, 1203–1216. doi: 10.1016/j.jmb.2006.04.027, PMID: 16677670

[ref30] Quemin E. Pietil M. K. Oksanen H. M. Forterre P. Rijpstra W. Schouten S.. (2015). Sulfolobus spindle-shaped virus 1 contains glycosylated capsid proteins, a cellular chromatin protein, and host-derived lipids. J. Virol. 89, 11681–11691. doi: 10.1128/JVI.02270-15, PMID: 26355093 PMC4645638

[ref31] Reuter M. Vestergaard G. Brügger K. Rachel R. Garrett R. Prangishvili D. (2005). Structure and genome organization of AFV2, a novel archaeal lipothrixvirus with unusual terminal and core structures. J. Bacteriol. 187, 3855–3858. doi: 10.1128/JB.187.11.3855-3858.2005, PMID: 15901711 PMC1112056

[ref32] Rice G. Stedman K. Snyder J. Wiedenheft B. Willits D. Brumfield S.. (2001). Viruses from extreme thermal environments. Proc. Natl. Acad. Sci. U. S. A. 98, 13341–13345. doi: 10.1073/pnas.231170198, PMID: 11606757 PMC60872

[ref33] Roine E. Bamford D. (2012). Lipids of archaeal viruses. Archaea 2012:384919. doi: 10.1155/2012/384919, PMID: 23049284 PMC3461281

[ref34] Sayers E. Beck J. Bolton E. Bourexis D. Brister J. Canese K.. (2021). Database resources of the national center for biotechnology information. Nucleic Acids Res. 49, D10–D17. doi: 10.1093/nar/gkaa892, PMID: 33095870 PMC7778943

[ref35] Söding J. Biegert A. Lupas A. (2005). The HHpred interactive server for protein homology detection and structure prediction. Nucleic Acids Res. 33, W244–W248. doi: 10.1093/nar/gki408, PMID: 15980461 PMC1160169

[ref07] Sprott D. Meloche M. Richards C. (1991). Proportions of diether, macrocyclic diether, and tetraether lipids in *Methanococcus jannaschii* grown at different temperatures. J. Bacteriol. 173, 3907–3910. doi: 10.1128/jb.173.12.3907-3910.1991, PMID: 2050642 PMC208025

[ref36] Wagner C. Reddy V. Asturias F. Khoshouei M. Johnson J. Manrique P.. (2017). Isolation and characterization of Metallosphaera turreted icosahedral virus (MTIV), a founding member of a new family of archaeal viruses. J. Virol. 91:925. doi: 10.1128/JVI.00925-17, PMID: 28768871 PMC5625487

[ref37] Wang F. Cvirkaite-Krupovic V. Vos M. Beltran L. Kreutzberger M. Winter J. M.. (2022). Spindle-shaped archaeal viruses evolved from rod-shaped ancestors to package a larger genome. Cells 185, 1297–1307.e11. doi: 10.1016/j.cell.2022.02.019, PMID: 35325592 PMC9018610

[ref38] Wang F. Liu Y. Su Z. Osinski T. de Oliveira G. Conway J.. (2019). A packing for A-form DNA in an icosahedral virus. Proc. Natl. Acad. Sci. 116, 22591–22597. doi: 10.1073/pnas.1908242116, PMID: 31636205 PMC6842630

[ref39] Wirth J. Munson-Mcgee J. H. Young M. J. (2021). Discovery of archaeal viruses in hot spring environments using viral metagenomics. Encycl. Virol. 19, 563–574. doi: 10.2166/wh.2021.101

[ref40] Xiang X. Chen L. Huang X. Luo Y. She Q. Huang L. (2005). *Sulfolobus tengchongensis* spindle-shaped virus STSV1: virus-host interactions and genomic features. J. Virol. 79, 8677–8686. doi: 10.1128/JVI.7914.8677-8686.2005, PMID: 15994761 PMC1168784

