# Supplementary material for: Isolation of archaeal viruses with lipid membrane from Tengchong acidic hot springs
Source: Front Microbiol. 2023 Mar 29;14:1134935. doi: 10.3389/fmicb.2023.1134935 (PMC10101205; doi:10.3389/fmicb.2023.1134935)
Supplement: Supplementary file 1 [file Data_Sheet_1.doc]

**Supplementary Material**

**Supplementary Tables**

**Table S1.** Sampling conditions and geological features of the 11 environmental samples collected in the solfataric field of Tengchong in Yunnan, China.

| **Sample** | **Coordinates** | **pH** | **Temperature (°C)** | **Features of hot spring** |
| --- | --- | --- | --- | --- |
| Drty-1 | 24°57'N 98°26'E | 2.92 | 67.7 | The sediment at the bottom of the spring is light gray, accompanied by a large number of leaves. |
| Drty-2 | 24°57'N 98°26'E | 2.82 | 64.8 | Pit is round and clear with a few bubbles in the lower part. |
| Drty-3 | 24°57'N 98°26'E | 2.32 | 72.9 | Pool is long and narrow, along the stone wall to the east and west, about 2 m in length and 0.5~1 m in width. |
| Drty-4 | 24°57'N 98°26'E | 2.29 | 74.1 | Pool is about 58 cm (length) × 24 cm (width) with bubbling. Sediment is brownish yellow in color. |
| Drty-5 | 24°57'N 98°26'E | 2.28 | 64.7 | Water is cloudy and algae are attached to the walls of the pit. |
| Drty-6 | 24°57'N 98°26'E | 2.23 | 70.9 | Water is cloudy and the sediment at the bottom of the spring is gray. |
| Drty-7 | 24°57'N 98°26'E | 2.10 | 70.7 | Many large bubbles and small bubbles, clear water. |
| Drty-8 | 24°57'N 98°26'E | 2.23 | 61.2 | Water is clear and sediments are pale gray |
| Drty-9 | 24°57'N 98°26'E | 2.52 | 56.3 | Pool has a "stomach" shape with a few bubbles and leaves. |
| Drty-10 | 24°57'N 98°26'E | 2.31 | 60.9 | The spring is huge and round, and the water is clear. |
| Zzq | 24°57' 4,.61''N 98°26' 9.12''E | 3.39 | 92.0 | Boiling spring. Creamy-white sandy sediment at the bottom, accompanied by more defoliation, with broad leaf and kidney fern. |

**Table S2.** Morphologies and number of the VLPs observed in enrichment cultures.

| Sample | Growth (OD_600_) | Morphologies and number of VLPs |
| --- | --- | --- |
| Drty1 | 1.23 | spherical +++* |
| Drty2 | 0.61 | rod-shaped and spherical + |
| Drty3 | 0.45 | lemon-shaped, rod-shaped and spherical +++++ |
| Drty4 | 0.50 | rod-shaped and spherical +++++ |
| Drty5 | 0.66 | lemon-shaped with tails and spherical ++ |
| Drty6 | 1.00 | no |
| Drty7 | 0.32 | no |
| Drty8 | 1.14 | rod-shaped and spherical + |
| Drty9 | 0.88 | lemon-shaped, rod-shaped and spherical ++ |
| Drty10 | 1.01 | spherical ++ |
| Zzq | 1.33 | lemon-shaped with tails and spherical ++ |

*The number of “+” signs indicate the number of VLPs that can be seen in a random TEM field.

**Table S3** The annotated ORFs of MTIV3, SIFV3 and STIV3.

| **ORF** | **Coordinates** | **Length aa** | **Annotation** | **BLAST hit** | **Identity (%), E-value** |
| --- | --- | --- | --- | --- | --- |
| MTIV3_ORF10 | 2481..2657 | 58 | CopG family transcriptional regulator | Metallosphaera sedula (MCP6729962.1) | 26/56(46%), 1e-04 |
| MTIV3_ORF13 | 3365..3949 | 194 | hypothetical protein b181 | Metallosphaera turreted icosahedral virus (ASO67406.1) | 51/194(26%), 2e-09 |
| MTIV3_ORF14 | 3909..4313 | 109 | hypothetical protein c109 | Metallosphaera turreted icosahedral virus (YP_009408151.1) | 39/104(38%), 9e-09 |
| MTIV3_ORF18 | 5441..5764 | 107 | virion structural protein | Metallosphaera turreted icosahedral virus (YP_009408155.1) | 30/69(43%), 2e-13 |
| MTIV3_ORF21 | 6493..6909 | 138 | structural protein c137 | Metallosphaera turreted icosahedral virus (YP_009408158.1) | 65/135(48%), 3e-41 |
| MTIV3_ORF22 | 6946..7668 | 240 | hypothetical protein c225 | Metallosphaera turreted icosahedral virus (YP_009408159.1) | 46/159(29%), 3e-07 |
| MTIV3_ORF23 | 7665..7964 | 99 | hypothetical protein b94 | Metallosphaera turreted icosahedral virus (YP_009408160.1) | 25/59(42%), 0.006 |
| SIFV3_ORF3 | 558..1178 | 206 | CRISPR/Cas system associated | Sulfolobus filamentous virus 6 (YP_001604166.1) | 83/198(42%), 2e-39 |
| SIFV3_ORF5 | 1750..1520 | 76 | hypothetical protein | Candidatus Aramenus sulfurataquae (MCL7344715.1) | 31/56(55%), 5e-13 |
| SIFV3_ORF7 | 2246..4120 | 624 | Holliday junction branch migration helicase | Acidianus filamentous virus 3 (YP_001604351.1) | 209/581(36%), 8e-128 |
| SIFV3_ORF17 | 8556..8882 | 108 | DUF1874 domain-containing protein | Sulfolobus islandicus rod-shaped virus 2 (NP_666571.1) | 45/94(48%), 4e-24 |
| SIFV3_ORF22 | 10131..10715 | 194 | hypothetical protein | Staphylococcus haemolyticus (WP_240570387.1) | 108/110(98%), 3e-71 |
| SIFV3_ORF24 | 10878 ..11480 | 200 | hypothetical protein | Staphylococcus haemolyticus (WP_240570386.1) | 173/173(100%), 8e-122 |
| SIFV3_ORF25 | 11532 ..12188 | 218 | virion structural protein | Sulfolobus islandicus filamentous virus (NP_445701.1) | 76/207(37%), 1e-31 |
| SIFV3_ORF26 | 12199 ..12687 | 162 | virion structural protein | Sulfolobus islandicus filamentous virus ( NP_445700.1) | 56/167(34%), 6e-29 |
| SIFV3_ORF27 | 12725..13000 | 91 | hypothetical protein | Candidatus Nanopusillus sp. (MCG2868371.1) | 33/81(41%), 3e-12 |
| SIFV3_ORF28 | 13000 ..13992 | 330 | glycosyltransferase | Sulfolobus islandicus filamentous virus (NP_445708) | 153/335(46%), 1e-96 |
| SIFV3_ORF29 | 14323..13997 | 108 | conserved lipothrixviral protein | Sulfolobus islandicus filamentous virus 2 (AOS58394.1) | 25/47(53%), 2e-07 |
| SIFV3_ORF30 | 14369 ..15160 | 263 | lipothrixviral glycosyltransferase | Sulfolobus islandicus filamentous virus 2 (AOS58392.1) | 101/260(39%), 3e-50 |
| SIFV3_ORF31 | 15161 ..15706 | 181 | conserved lipothrixviral protein | Sulfolobus islandicus filamentous virus 2 (AOS58385.1) | 67/157(43%), 3e-42 |
| SIFV3_ORF32 | 15757 ..15972 | 71 | hypothetical protein AFV7_gp37 | Acidianus filamentous virus 7 (YP_001604261.1) | 60/71(85%), 2e-32 |
| SIFV3_ORF33 | 16013 ..16432 | 139 | conserved lipothrixviral protein | Sulfolobus islandicus filamentous virus 2 (AOS58384.1) | 35/97(36%), 7e-12 |
| SIFV3_ORF34 | 16429 ..17070 | 213 | nucleotidyltransferase | Sulfolobus sp. (PVU70774.1) | 102/221(46%), 7e-46 |
| SIFV3_ORF35 | 17707 ..17057 | 216 | hypothetical protein HOT91_gp41 | Sulfolobus filamentous virus 1 (YP_009808151.1) | 48/146(33%), 4e-04 |
| SIFV3_ORF38 | 21132..19483 | 549 | hypothetical protein SBFV1_gp51 | Sulfolobales Beppu filamentous phage 1 (AZI75752.1) | 120/486(25%), 2e-29 |
| SIFV3_ORF40 | 22967..21936 | 343 | hypothetical protein HOT91_gp48 | Sulfolobus filamentous virus 1 (YP_009808158.1) | 132/354(37%), 5e-39 |
| SIFV3_ORF43 | 24316 ..24047 | 89 | hypothetical protein HOT91_gp44 | Sulfolobus filamentous virus 1 (YP_009808154.1) | 30/93(32%), 2e-07 |
| SIFV3_ORF45 | 24889 ..24590 | 99 | hypothetical protein HOT91_gp38 | Sulfolobus filamentous virus 1 (YP_009808148.1) | 31/95(33%), 6e-05 |
| SIFV3_ORF48 | 25587 ..25973 | 128 | hypothetical protein AFV9_gp35 | Acidianus filamentous virus 9 (YP_001798553.1) | 33/62(53%), 1e-12 |
| SIFV3_ORF50 | 26566 ..26195 | 123 | hypothetical protein | Staphylococcus haemolyticus (WP_240570378.1) | 64/64(100%), 4e-36 |
| SIFV3_ORF51 | 26659 ..27486 | 275 | hypothetical protein | Staphylococcus haemolyticus (WP_240570377.1) | 257/260(99%),0 |
| SIFV3_ORF53 | 27490 ..28392 | 300 | hypothetical protein | Staphylococcus haemolyticus (WP_240570376.1) | 239/243(98%), 4e-174 |
| SIFV3_ORF54 | 28415..28666 | 83 | hypothetical protein | Acidilobus sp. (NAZ31811.1) | 32/79(41%), 4e-06 |
| SIFV3_ORF55 | 29335..28667 | 222 | hypothetical protein CCL43_gp46 | Sulfolobus islandicus rod-shaped virus 7 (YP_009362546.1) | 78/174(45%), 2e-39 |
| STIV3_ORF5 | 2334..1651 | 227 | hypothetical protein c225 | Metallosphaera turreted icosahedral virus (YP_009408159.1) | 50/155(32%), 8e-04 |
| STIV3_ORF6 | 2781..2368 | 137 | structural protein c137 | Metallosphaera turreted icosahedral virus (YP_009408158.1) | 81/136(58%), 2e-50 |
| STIV3_ORF8 | 3367..3107 | 86 | virion structural protein | Metallosphaera turreted icosahedral virus (YP_009408156.1) | 31/82(39%), 3e-10 |
| STIV3_ORF9 | 3649..3371 | 92 | virion structural protein | Metallosphaera turreted icosahedral virus (YP_009408155.1) | 27/64(42%), 8e-08 |
| STIV3_ORF13 | 4835..4452 | 127 | hypothetical protein c109 | Metallosphaera turreted icosahedral virus (YP_009408151.1) | 44/103(43%), 8e-18 |
| STIV3_ORF27 | 10935..10624 | 103 | pyramid forming protein | Stygiolobus rod-shaped virus (YP_009094259.1) | 46/101(46%), 2e-12 |
| STIV3_ORF30 | 12766..12098 | 222 | hypothetical protein | Candidatus Nanopusillus sp. (MCG2868117.1) | 77/207(37%), 4e-19 |
| STIV3_ORF33 | 13896..13702 | 64 | structural protein c137 | Metallosphaera turreted icosahedral virus (YP_009408158.1) | 39/63(62%), 5e-21 |
| STIV3_ORF35 | 14702..14424 | 92 | hypothetical protein CBR30_09750 | Dictyoglomus sp. (PMQ00705.1) | 31/73(42%), 8e-09 |
| STIV3_ORF36 | 15030..14683 | 115 | virion structural protein | Metallosphaera turreted icosahedral virus (YP_009408155.1) | 32/80(40%), 1e-17 |
| STIV3_ORF40 | 16405 ..15917 | 162 | hypothetical protein c109 | Metallosphaera turreted icosahedral virus (ASO67407.1) | 38/107(36%), 5e-10 |
| STIV3_ORF45 | 17553..17392 | 53 | DUF6290 family protein | Candidatus Nanopusillus sp. (MCG2868119.1) | 19/42(45%), 0.001 |
| STIV3_ORF46 | 17813..17550 | 87 | hypothetical protein AXI69_gp01 | Sulfolobus monocaudavirus SMV3 (YP_009226224.1) | 24/65(37%), 2e-04 |

**Supplementary Figures**


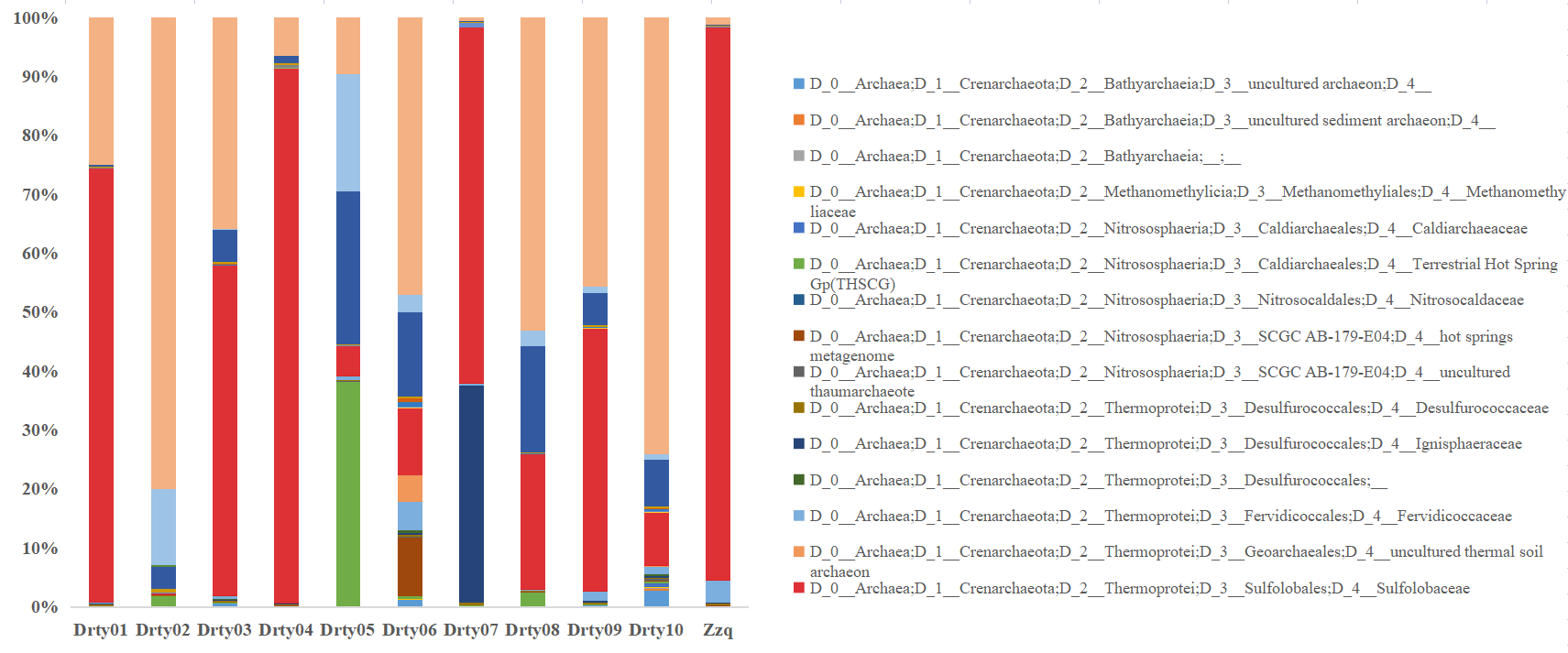


**Figure S1**. Taxonomic composition of 16S rRNA gene amplicon sequences obtained from hot spring sediments. The shown taxa have >0.1% relative abundance in at least one single sample. The red columns represent family *Sulfolobaceae*.


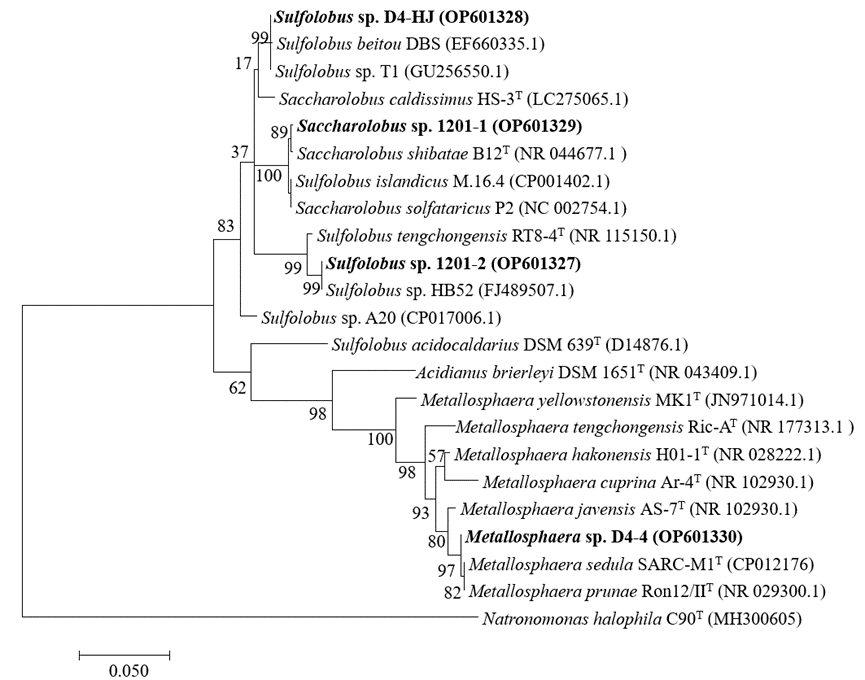


**Figure S2.** A 16S rRNA gene-based phylogenetic tree of host strains and related groups. The percentages of replicate trees in which the associated taxa clustered together in the bootstrap test (1,000 replicates) are shown next to the branches. The evolutionary distances are in units of the number of base substitutions per site.
